# Placental *OPRM1* DNA methylation and associations with neonatal opioid withdrawal syndrome, a pilot study

**DOI:** 10.37349/emed.2020.00009

**Published:** 2020-06-29

**Authors:** Elisha M. Wachman, Alice Wang, Breanna C. Isley, Jeffery Boateng, Jacob A. Beierle, Aaron Hansbury, Hira Shrestha, Camron Bryant, Huiping Zhang

**Affiliations:** 1 Department of Pediatrics, Boston Medical Center, Boston, MA 02119, USA; 2 Boston University School of Public Health, Boston, MA 02118, USA; 3 Laboratory of Addiction Genetics, Department of Pharmacology and Experimental Therapeutics and Psychiatry, Boston University School of Medicine, Boston, MA 02118, USA; 4 Department of Psychiatry, Boston University School of Medicine, Boston, MA 02118, USA

**Keywords:** neonatal opioid withdrawal syndrome, neonatal abstinence syndrome, DNA methylation, *OPRM1*, epigenetics, placenta, opioids

## Abstract

**Aims:**

Epigenetic variation of DNA methylation of the mu-opioid receptor gene (*OPRM1*) has been identified in the blood and saliva of individuals with opioid use disorder (OUD) and infants with neonatal opioid withdrawal syndrome (NOWS). It is unknown whether epigenetic variation in *OPRM1* exists within placental tissue in women with OUD and whether it is associated with NOWS outcomes. In this pilot study, the authors aimed to 1) examine the association between placental *OPRM1* DNA methylation levels and NOWS outcomes, and 2) compare *OPRM1* methylation levels in opioid-exposed *versus* non-exposed control placentas.

**Methods:**

Placental tissue was collected from eligible opioid (*n* = 64) and control (*n* = 29) women after delivery. Placental DNA was isolated and methylation levels at six cytosine-phosphate-guanine (CpG) sites within the *OPRM1* promoter were quantified. Methylation levels were evaluated for associations with infant NOWS outcome measures: need for pharmacologic treatment, length of hospital stay (LOS), morphine treatment days, and treatment with two medications. Regression models were created and adjusted for clinical co-variates. Methylation levels between opioid and controls placentas were also compared.

**Results:**

The primary opioid exposures were methadone and buprenorphine. Forty-nine (76.6%) of the opioid-exposed infants required pharmacologic treatment, 10 (15.6%) two medications, and average LOS for all opioid-exposed infants was 16.5 (standard deviation 9.7) days. There were no significant associations between *OPRM1* DNA methylation levels in the six CpG sites and any NOWS outcome measures. No significant differences were found in methylation levels between the opioid and control samples.

**Conclusions:**

No significant associations were found between *OPRM1* placental DNA methylation levels and NOWS severity in this pilot cohort. In addition, no significant differences were seen in *OPRM1* methylation in opioid *versus* control placentas. Future association studies examining methylation levels on a genome-wide level are warranted.

## Introduction

Incidence of neonatal opioid withdrawal syndrome (NOWS), or neonatal abstinence syndrome (NAS), increased over four-fold from 2004 to 2014 [1]. After birth, infants at risk for this highly variable syndrome are observed in-hospital for signs of opioid withdrawal, which typically begin at 2–3 days of life. Anywhere from 10–80% of these infants receive pharmacological treatment, typically morphine or methadone, with an average length of hospital stay of 2–3 weeks resulting in significantly higher associated hospitalization costs compared to their non-opioid exposed counterparts [2, 3].

Many clinical factors contribute to variability in NOWS outcomes. Although the dose of the maternal opioid agonist medication the infant is exposed to does not predict NOWS severity, factors such as type of maternal opioid medication (methadone or buprenorphine), the infant’s gestational age (GA) and sex, breastfeeding, non-pharmacologic care, and concurrent exposures to psychiatric medications, nicotine, or illicit drugs have been associated with differences in NOWS severity [4–7]. Despite this knowledge of clinical variables, accurately predicting the disease course for each infant remains futile.

In recent years, both genetic and epigenetic factors have been identified in association with differences in NOWS outcomes [8]. Single nucleotide polymorphisms (SNPs) in the mu-opioid receptor gene (*OPRM1*), as well as additional opioid and stress response genes have been identified in both mothers and infants with opioid-exposure in relation to differences in NOWS severity [9–11]. In addition, associations between increased cytosine-phosphate-guanine (CpG) dinucleotide methylation within the *OPRM1* gene promoter region and NOWS outcomes have been observed, with increased methylation at select CpG sites associated with more severe NOWS [12, 13].

The placenta, a temporary organ during pregnancy that represents the link between the mother and the fetus, is a key target for epigenetic modification as the master regulator of the fetal environment [14]. The placenta is uniquely sensitive to environmental influences and epigenetic modification, and subsequent differences in gene expression and tissue differentiation [15–17]. Triggers of such epigenetic modification can include prenatal exposure to maternal psychosocial stress, nicotine, and illicit drugs [18–21]. Previous studies have shown an association between placental DNA methylation in key genes after exposure to maternal stress and differences in infant neurobehavior [22, 23]. In terms of in-utero substance exposure, exposure to nicotine has been associated with epigenetic variation and low birth weight [14, 20]. To our knowledge, the influence of in-utero exposure to prescribed opioids such as methadone and buprenorphine on placental DNA methylation has not been previously examined.

Given these associations of placental epigenetic modifications to infant neurobehavior following in utero exposure to other substances, and based on our promising results from previous studies on *OPRM1* in relation to NOWS [9–13], we sought to examine the association between placental *OPRM1* DNA methylation and infant NOWS outcomes, and to compare the level of placental *OPRM1* methylation in opioid-exposed *versus* non-exposed subjects. Implications of this research would be an understanding of how maternal opioid exposure modifies this important maternal-fetal interface and potentially impacts NOWS presentation.

## Materials and methods

### Setting

Boston Medical Center (BMC) is the largest urban safety net hospital in New England with a specialized prenatal clinic for women with opioid use disorder (OUD), Project RESPECT. Treatment options for pregnant women with OUD include methadone and buprenorphine. BMC practices a rooming-in model of care where infants room-in with their mothers in the postpartum room until maternal discharge, and then the infants are transferred to the pediatric inpatient unit for continued NOWS monitoring and treatment, where their mothers can continue to room-in.

### NOWS guidelines

All opioid-exposed infants were cared for according to routine hospital care guidelines for NOWS. Infants with known prenatal opioid exposure are monitored for 5–7 days for NOWS that may require pharmacologic treatment. During the study period, infants were assessed with the original Finnegan scale every 4 h, with the criteria for initiation and escalation of medication being 2 scores ≥ 8 or 1 score ≥ 12 [24, 25]. First-line pharmacologic treatment during the study period was neonatal morphine solution which was titrated until scores were < 8, then weaned by 10% of the maximum daily dose as often as once every day until the infant was down to 20% of the maximum dose, then stopped. Infants were monitored for a minimum of 24 h off medication prior to discharge. Second line treatment was initiated if infants received maximum recommended doses of morphine with continued elevated Finnegan scores ≥ 8 or were stalled in their wean and consisted of phenobarbital or clonidine depending on prenatal exposures. Clonidine was weaned in the inpatient setting after weaning off morphine, while phenobarbital was weaned as an outpatient by 20% per week starting 48 h after morphine was stopped. This strict NOWS management protocol was in place for the duration of the study with interrater reliability for Finnegan scores of ≥ 0.80 [25]. Mothers were eligible to provide breastmilk if they had received adequate prenatal care, were in an addiction treatment program, and had no recent illicit drug use close to the time of delivery [26].

### Subjects

This study was approved by the Boston University Medical Campus Institutional Review Board. Subjects were enrolled between October 2013 and October 2017. Written consent was obtained only from those subjects in the opioid cohort as part of a more extensive NOWS genetics study protocol that focused on maternal and infant saliva DNA collection for genetic and epigenetic analyses [9, 13]. Eligibility criteria for inclusion in the opioid cohort included prenatal care in BMC’s Project RESPECT clinic, maternal treatment with prescribed methadone or buprenorphine for at least 30 days prior to delivery, no major social or psychiatric concerns that would alter ability to participate in research and provide informed consent, singleton pregnancies, gestational age of ≥ 36 weeks, and delivery at BMC. Mothers were recruited prenatally in the second and third trimesters from Project RESPECT or consented shortly after delivery on the postpartum unit.

Informed consent was waived for those subjects in the control group due to the collection of discarded placental tissue only for these subjects along with limited de-identified basic demographic data from electronic medical record (EMR). The EMRs for these subjects were accessed once at the time of placental collection, with no identifiers or master code collected per IRB guidelines. For the control group, eligibility criteria for placental collection included delivery at BMC, GA ≥ 36 weeks, and absence of a known substance use disorder per the EMR problem list, admission note, and review of toxicology screen lab results.

For the final analyses, only placental epigenetic data of controls with no EMR-documented smoking history and/or other significant health problems, that are known to alter placental health (e.g., pre-eclampsia), were included. These controls were matched with an equal number of subjects on methadone and buprenorphine based on the month of delivery. Health problems that alter placental health were also an exclusion factor for analysis for the opioid cohort.

### Phenotype data collection

For the opioid cohort, extensive chart reviews were performed using the electronic health record. Baseline maternal characteristics were collected including ethnicity and race, methadone *versus* buprenorphine treatment for OUD, additional co-exposures (psychiatric medications, illicit drugs, and nicotine in the third trimester), maternal age, and pregnancy complications and outcomes. For the opioid-exposed infants, we collected birth parameters and complications, breastfeeding (defined as any amount of breast milk consumed by the infant during the hospitalization), and details of NOWS treatment and hospitalization including medication treatment and length of hospital stay (LOS). Limited data points were collected for the control group at the time of placental collection, including maternal ethnicity, maternal age, smoking status, gestational age at delivery, infant birth weight, and infant sex. All data were hand abstracted and entered into an electronic database. Data was checked for accuracy and missingness with any discrepancies addressed prior to data analysis.

Placental tissue collection and *OPRM1* promoter DNA methylation assay: a placental sample was collected within 2 h of delivery from all subjects from the Labor and Delivery Unit at BMC. A sample of approximately 1 inch × 1 inch in size was collected from the maternal side of the placenta with the goal of collecting the villous tissue using forceps after removing the outer chorion layer. The sample was placed in a 50 mL conical tube, then frozen at −80 degrees Celsius within 2 h of delivery. Genomic DNA was isolated from frozen placental tissue using Qiagen DNeasy columns per standard protocols. Purified DNA was sent to EpigenDx, Inc. (Hopkinton, MA, USA; https://www.epigendx.com/) for *OPRM1* promoter DNA methylation assay that was described below.

For DNA methylation analysis, 250 ng of extracted genomic DNA was bisulfite treated using the EZ DNA Methylation kit (Zymo Research, Inc., CA) (See Supplemental Methods). A CpG island was located 1, 000 nucleotides downstream of the transcription start site of *OPRM1*. CpG sites were labeled relative to the A of the ATG translation start site. A total of 6 CpG dinucleotides, located at −60, −50, −32, −25, −18 and −14 degrees, were examined. The methylation status of each CpG site was determined individually as an artificial C/T SNP using QCpG software (Pyrosequencing, Qiagen). The percent methylation level at each CpG site was calculated as the number of methylated alleles divided by the sum of all methylated and unmethylated alleles times one hundred. The mean methylation level was calculated using methylation levels of all measured CpG sites within the targeted region of each gene. Each experiment included non-CpG cytosines as internal controls to detect incomplete bisulfite conversion of the input DNA. In addition, a series of unmethylated and methylated DNAs were included as controls in each PCR. Furthermore, PCR bias testing was performed by mixing unmethylated control DNA with *in vitro* methylated DNA at different ratios (0%, 5%, 10%, 25%, 50%, 75%, and 100%), followed by bisulfite modification, PCR, and pyrosequencing analysis.

### Statistical methods

Outcome measures for NOWS severity included 1) any NOWS pharmacologic treatment (yes/no), 2) treatment with two medications for NOWS (yes/no), 3) LOS in days due to NOWS [defined as 48 h after stopping NOWS inpatient medications (morphine and/or clonidine), or 24 h after stopping NOWS scoring if hospitalization were prolonged for social circumstances or other medical complications], and 4) morphine treatment days.

Baseline demographics for the opioid and control cohorts were summarized. NOWS outcomes were compared using *t*-tests and the correlation analysis was based on the level of methylation at each of the 6 CpG sites and the mean methylation level per placenta. Then, potential covariates and confounders that could influence NOWS outcomes were examined including maternal opioid type, co-exposure to nicotine, co-exposure to psychiatric medications, infant sex, and breastfeeding. Variables were chosen for examination based on established prior literature showing associations between these variables and NOWS outcomes. We also assessed for associations between DNA methylation and maternal smoking, a known trigger for methylation differences [20]. An alpha level of 0.05 was used for inclusion in regression models. Data was assessed for normality. Multivariate linear and logistic regression models were created for each of the NOWS outcomes including the significant covariates. For categorical outcome variables (i.e. NOWS treatment, treatment with 2 medications), adjusted odds ratios were obtained, and reported as the odds of the outcome based on a 1% increase in the level of DNA methylation at that CpG site. For continuous outcome measures (LOS, morphine treatment days), beta-values were reported corresponding to the change in the outcome measure in days with every 1% increase in methylation level at the CpG site. SAS version 9.4 was used for all analyses. An alpha level of 0.05 was used for point-wise significance. Lastly, we applied the Benjamini-Hochberg method to account for multiple testing of 6 CpG sites with an alpha value of 0.008 for experiment-wise significance [27]. No sample power calculation was performed due to the pilot nature of this study.

## Results

During the study time period of October 2013 to October 2017, placental samples were collected from 84 opioid-exposed subjects, of which 64 were included in this analysis (29 on methadone and 35 on buprenorphine). The remaining 20 samples were banked for future analysis given the pilot nature of this study. For control samples, 45 samples were collected, of which 29 met our analysis criteria of being non-smokers with GA ≥ 36 weeks and no major concurrent maternal illnesses that could impact placental health.

There were no significant differences in the baseline demographics between the opioid and control cohorts with the exception of differences in maternal ethnicity, with more white non-Hispanics in the opioid cohort, consistent with prior publications in this patient population (Table 1) [28, 29].

For the opioid cohort, almost half of the mothers were also using nicotine in the third trimester, and a third had concurrent illicit drug exposure to cocaine, cannabinoids, heroin, or other illicit opioids. Forty-nine (76.5%) of the infants required pharmacologic treatment, 10 (15.6%) were treated with two medications (7 with phenobarbital and 3 with clonidine), with an average LOS for all opioid exposed infants of 16.5 (SD 9.7) days (Table 2).

**Figure 1.**
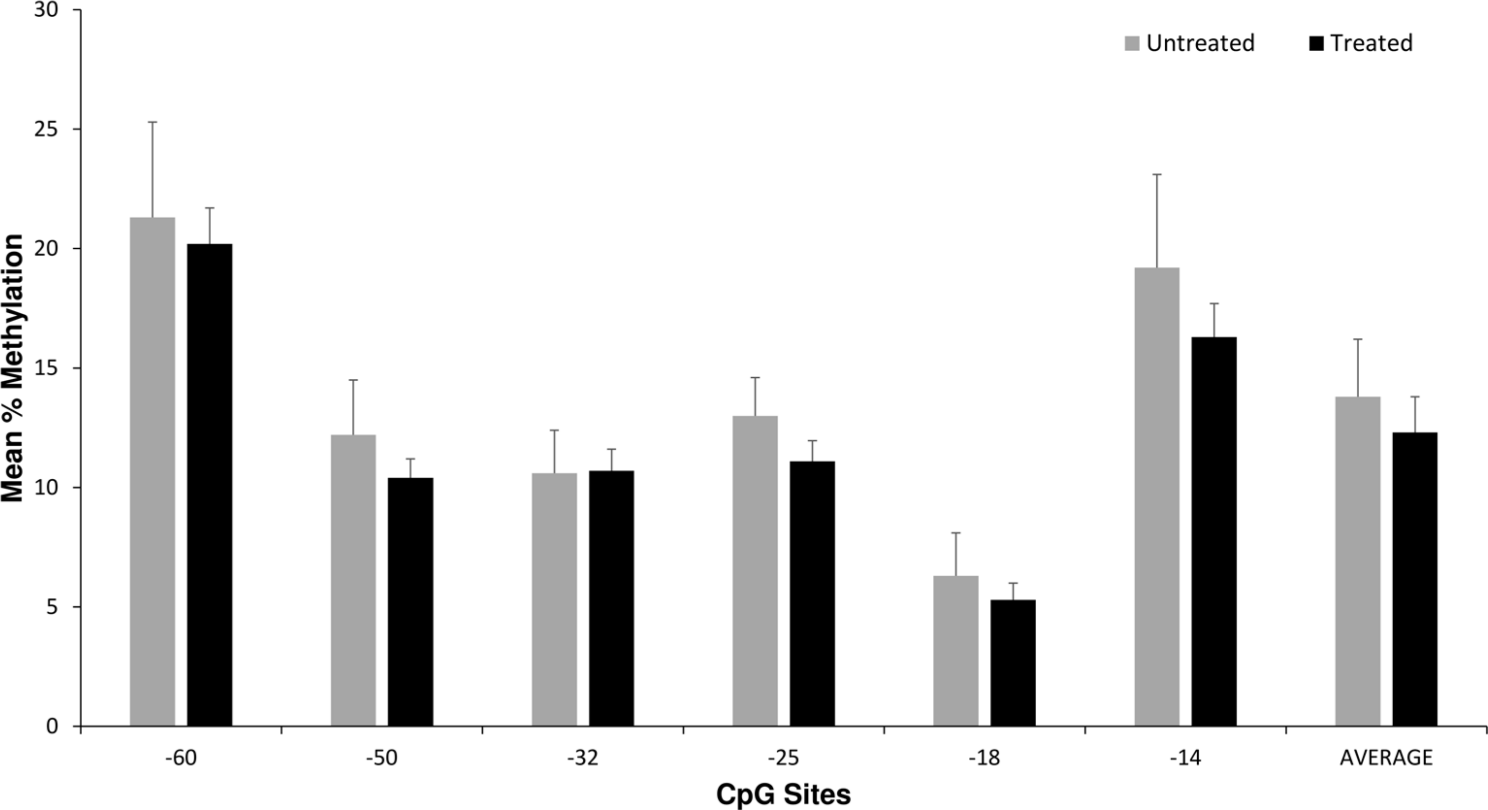
Placenta *OPRM1* DNA methylation and NOWS pharmacologic treatment. Average placental *OPRM1* methylation levels for CpG sites for the 64 opioid subjects based on NOWS pharmacologic treatment (*n* = 49) *versus* no treatment (*n* = 15). There were no statistically significant differences in univariate or multivariate analyses

There were no significant associations between *OPRM1* DNA methylation levels in the 6 CpG sites or the average level of methylation per subject with any NOWS outcome measures (Figure 1 and Table 3). In covariate association analyses, maternal opioid medication type (methadone *versus* buprenorphine), maternal smoking, and illicit drug exposure were most strongly associated with differences in the primary outcome measures of NOWS pharmacologic treatment and LOS (*P* < 0.05 for all, data not shown). The opioid cohort was also tested for associations between methylation levels and maternal smoking status and no associations were found. In linear and logistic regression models adjusting for illicit drugs (for outcome measures LOS and morphine treatment days), maternal smoking (for all outcome measures), and maternal opioid type (for LOS, morphine treatment days, and NOWS pharmacologic treatment), no significant associations were identified (Table 3).

**Figure 2.**
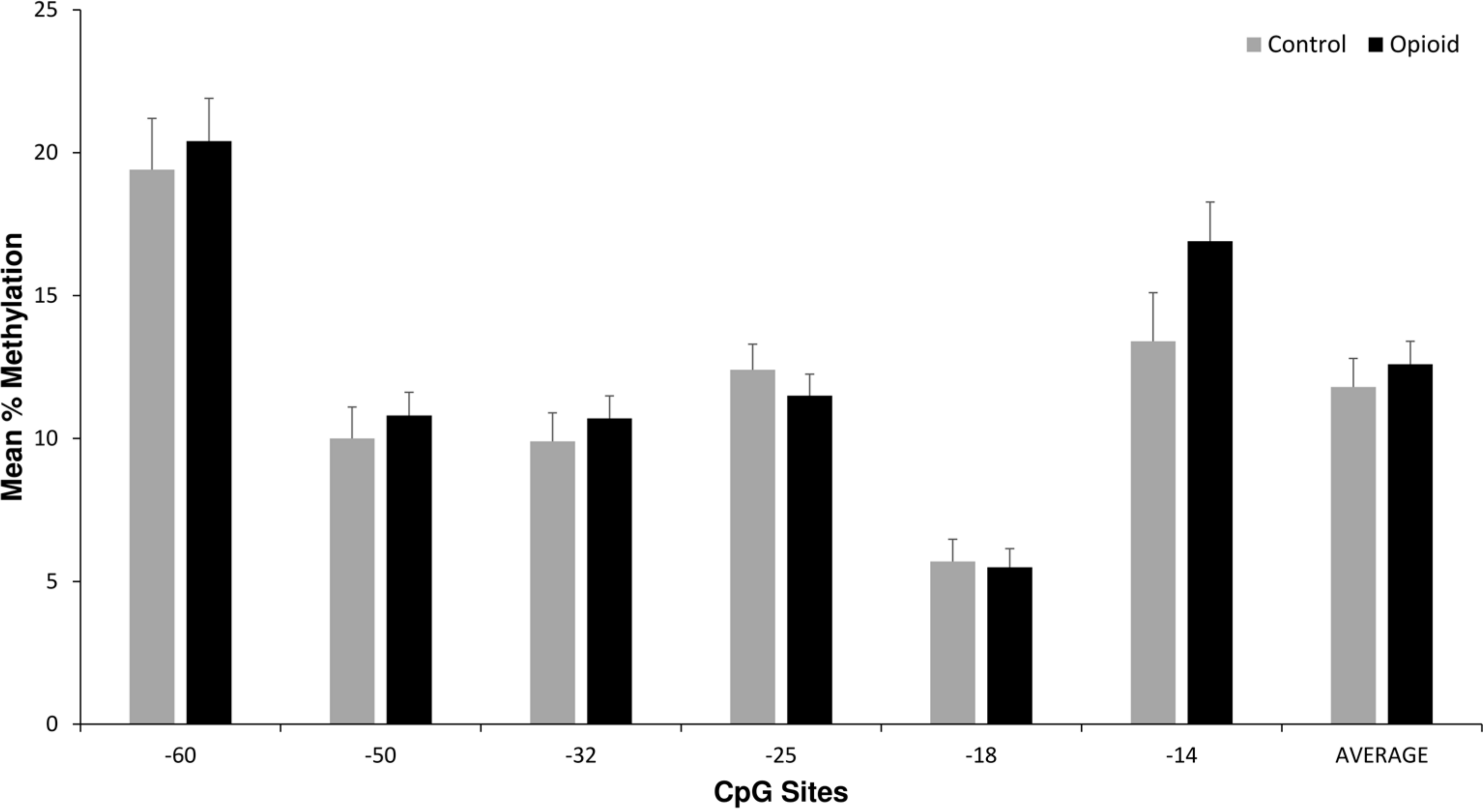
Placenta *OPRM1* DNA methylation in control *versus* opioid subjects. Average placental *OPRM1* methylation levels for CpG sites for the 64 opioid subjects compared with the 29 control subjects. There were no statistically significant differences in univariate or multivariate analyses

There were no significant differences in methylation levels at any of the CpG sites or the average methylation level per subject between the opioid and control subjects (Figure 2). A subset analysis was conducted looking at controls (*n* = 29, all non-smokers) and non-smoking opioid subjects (*n* = 20). There were also no significant differences in *OPRM1* methylation levels at any CpG site or the average methylation level between the two groups of subjects (data not shown).

## Discussion

In this pilot study, we found no significant associations between placental *OPRM1* DNA methylation levels and NOWS outcome measures. We also did not observe a significant difference in placental *OPRM1* DNA methylation levels between opioid and control subjects. These negative results could be explained by a small sample size. Alternatively, *OPRM1* DNA methylation in the placenta may not influence NOWS outcomes, emphasizing the need for further studies examining other genes in a larger sample as well as genome-wide differential methylation.

*OPRM1* is a primary candidate gene that has been examined in association with OUD and other addictive behaviors such as alcohol use disorder, with both genetic and epigenetic associations identified [30, 31]. In non-pregnant adults, increased DNA methylation in the promoter region of *OPRM1* was associated with decreased expression of the gene, increased rates of opioid dependence, and higher doses of postoperative pain medications [31–35]. Similarly, our two prior studies of *OPRM1* DNA methylation in opioid-exposed pregnancies uncovered associations between increased methylation levels at key CpG sites and increased need for pharmacotherapy for NOWS in mother and infant saliva [12, 13]. Sample sizes in those studies were slightly larger (*n* = 86 and *n* = 113 respectively), with a different cell-type source (saliva, buccal cell, and cord blood). DNA methylation patterns are known to vary depending on the cell type and tissue source. The human placenta has lower global DNA methylation levels compared with other tissues, e.g., somatic. The placenta is heterogeneous with many cell types, and each may exhibit different epigenetic features [15]. Single cell methodologies could test whether NOWS is associated with differential *OPRM1* methylation in specific cell types of the placenta.

The placenta is the primary interface between the mother and the fetus and is uniquely sensitive to environmental exposures that can alter placental function and fetal development [16]. Epigenetic changes are known to be influenced by maternal life context factors, including diet, toxic exposures such as opioids and nicotine, and stress [20, 36]. The fetus is highly susceptible to epigenetic modification during development, which can have lasting effects on neurodevelopment [36]. Prior studies in non-opioid exposed pregnancies have found associations between placental epigenetic variation and infant neurobehavioral changes. Specifically, prenatal stress has been linked with higher levels of placental DNA methylation in the glucocorticoid receptor gene (*NR3C1*) and un-coordinated stress response in the infant. Conversely, maternal stress has been associated with lower levels of placental DNA methylation in the hydroxysteroid 11-beta dehydrogenase 2 gene (*HSD11B2*), and better stress tolerance [23, 37, 38].

Novel aspects of this study include examination of epigenetic variation in placental tissue in opioid-exposed pregnancies and *OPRM1* DNA methylation specifically within placental tissue. It also demonstrated the feasibility of obtaining adequate placental tissue from this high-risk population for epigenetic evaluation. In addition, the NOWS outcome measures were standardized and consistent with the clinical phenotypes presented in prior NOWS genetic association studies [8].

This study has a number of limitations. First, it is limited by small sample size that was underpowered to detect small differences in methylation levels. Also, there was racial and ethnic variability between the two groups which could have impacted methylation levels and placental mediated pregnancy complications; future studies should match samples by ethnicity. Exposure to multiple drugs including psychiatric medications and nicotine which is the norm for those with OUD made the association analysis more challenging as other drugs can also alter DNA methylation levels. In addition, we did not perform detailed questionnaires on exposures and other environmental epigenetic triggers such as stress which could be pursued in future larger studies. NOWS treatment protocols also vary between hospitals, thus replication of these findings in other settings would be important. Lastly, there were limitations of the control population with limited knowledge of other demographic variables to allow for better matching with the opioid subjects.

This pilot work is the first step to further examining epigenetic variation in placental tissue within opioid-exposed pregnancies, and potential importance for neonatal outcomes and the transgenerational risk. Next steps could include looking at a larger sample and examining genes involved in stress responses, quantifying methylation of other opioid receptor genes, or to look at methylation on a genome-wide level [16, 17, 39]. In addition, the use of contemporary genomics techniques such as RNA-seq has allowed us to analyze the adaptive cellular transcriptome in response to certain stimuli (for example, psychosocial stress or chronic drug treatment) and identify genomic regions underlying variation in behavioral traits [40]. Examining the correlation between genes that are both differentially methylated and expressed between groups of individuals can help to target key candidate genes underlying this risk [40–41].

In conclusion, this was a pioneering study examining placental epigenetic variation in *OPRM1* within opioid-exposed dyads. Though no associations were found, the placental study is a proof-of-concept for examining the link between maternal opioid exposure, epigenetic variation, and NOWS outcomes in the developing infant. Future larger scale studies should examine additional placental epigenetic markers and gene expression, along with additional infant neurobehavioral outcomes.

## Supplementary Material

Supplementary Material

## Figures and Tables

**Table 1.** Demographics of control and opioid mother-infant dyads

Demographic	Controls (*n* = 29)	OPIOID (*n* = 64)	*P*-value
	Mean (SD) or *n* (%)	Mean (SD) or *n* (%)	

Maternal age (years)	30.8 (6.0)	29.1 (5.3)	0.18
Infant gestational age (weeks)	39.1 (1.4)	39.0 (1.7)	0.73
Infant birth weight (grams)	3313.5 (486.7)	3134.8 (516.4)	0.12
Cesarean delivery	9 (31.0%)	25 (39.1%)	0.46
Infant sex = male	16 (55.2%)	29 (45.3%)	0.38
Maternal ethnicity			
White non-Hispanic	12 (41.4%)	56 (87.5%)	< 0.0001
Hispanic	1 (3.5%)	0	
Black	14 (48.3%)	5 (7.8%)	
Other	2 (6.9%)	3 (4.7%)	

SD: standard deviation

**Table 2.** Maternal exposures and NOWS outcomes of the opioid cohort

Demographic	Mean (SD) or *n* (%)

Maternal OUD treatment	
Methadone	29 (45.3%)
Buprenorphine	35 (54.7%)
Dose at delivery (mg/day)	
Methadone	80.5 (35.4)
Buprenorphine	11.4 (6.3)
Breastfed	32 (50.0%)
Co-exposures	
Nicotine smoking	44 (47.3%)
SSRIs	1 (1.6%)
Benzodiazepines	7 (11.1%)
Illicit drugs	35 (37.6%)
NOWS pharmacologic treatment	49 (76.6%)
Two pharmacologic agents for NOWS	10 (15.6%)
LOS for NOWS (days)	16.5 (9.7)
NOWS morphine treatment days	12.4 (8.8)

**Table 3.** Association of placental *OPRM1* methylation levels with NOWS outcomes

CpG site	Unadjusted results	*P*-value univariate	Adjusted results	*P*-value multivariate

**NOWS pharmacologic treatment: OR (95% CI)**				
−60	1.00 (0.95–1.06)	0.92	1.01 (0.95–1.07)	0.83
−50	0.98 (0.89–1.07)	0.62	0.97 (0.87–1.08)	0.52
−32	1.01 (0.92–1.11)	0.86	1.02 (0.90–1.16)	0.75
−25	0.97 (0.87–1.08)	0.53	0.96 (0.84–1.09)	0.50
−18	0.99 (0.88–1.11)	0.84	1.04 (0.90–1.19)	0.62
−14	0.99 (0.93–1.05)	0.67	0.99 (0.93–1.06)	0.76
Average	0.99 (0.89–1.09)	0.78	0.99 (0.89–1.11)	0.90
**LOS (days) due to NOWS: beta (95% CI)**				
−60	−0.001 (−0.19, 0.19)	0.99	−0.02 (−0.20, 0.17)	0.85
−50	0.03 (−0.30, 0.37)	0.84	0.02 (−0.29, 0.34)	0.89
−32	0.007 (−0.33, 0.34)	0.99	−0.06 (−0.39, 0.27)	0.72
−25	−0.04 (−0.41, 0.32)	0.81	−0.08 (−0.43, 0.20)	0.66
−18	−0.16 (−0.58, 0.27)	0.47	−0.05 (−0.47, 0.38)	0.83
−14	0.06 (−0.14, 0.26)	0.55	0.07 (−0.13, 0.27)	0.48
Average	0.01 (−0.33, 0.36)	0.94	0.0009 (−0.33, 0.33)	0.99
**Morphine treatment days: beta (95% CI)**				
−60	1.00 (0.94, 1.07)	0.92	1.00 (0.94, 1.07)	0.97
−50	0.96 (0.86, 1.07)	0.47	0.94 (0.84, 1.06)	0.35
−32	1.07 (0.96, 1.18)	0.22	1.05 (0.95, 1.17)	0.34
−25	1.05 (0.93, 1.19)	0.43	1.03 (0.92, 1.17)	0.59
−18	0.99 (0.87, 1.14)	0.98	0.99 (0.86, 1.14)	0.90
−14	1.01 (0.94, 1.08)	0.83	1.00 (0.94, 1.07)	0.98
Average	1.02 (0.97, 1.13)	0.77	1.01 (0.90, 1.12)	0.92
**Two medications to treat NOWS: OR (95% CI)**				
−60	−0.06 (−0.27, 0.14)	0.54	−0.05 (−0.26, 0.16)	0.62
−50	0.06 (−0.29, 0.42)	0.72	0.07 (−0.29, 0.44)	0.69
−32	−0.13 (−0.48, 0.21)	0.44	−0.14 (−0.51, 0.23)	0.45
−25	−0.06 (−0.42, 0.30)	0.73	−0.06 (−0.44, 0.32)	0.74
−18	−0.16 (−0.64, 0.32)	0.51	−0.17 (−0.69, 0.34)	0.50
−14	0.05 (−0.17, 0.28)	0.63	0.07 (−0.16, 0.29)	0.56
Average	−0.05 (−0.44, 0.33)	0.78	−0.04 (−0.44, 0.36)	0.85

NOWS pharmacologic treatment adjusted for smoking and maternal opioid; LOS and morphine treatment days adjusted for illicit drugs, smoking, and maternal opioid; two medications for NOWS adjusted for smoking. CI: confidence interval; OR: odds ratio

## Data Availability

All relevant data is contained within the manuscript. The dataset used for this manuscript will not be made publicly available due to the confidentiality issues and the sensitive nature of the data related to maternal substance use disorder.

## Abbreviations

CI: confidence interval
CpG: cytosine-phosphate-guanine
BMC: Boston Medical Center
EMR: electronic medical record
GA: gestational age
LOS: length of hospital stay
NAS: Neonatal Abstinence Syndrome
NOWS: neonatal opioid withdrawal syndrome
OPRM1: mu-opioid receptor gene
OR: odds ratio
OUD: opioid use disorder
SD: standard deviation
SNPs: single nucleotide polymorphisms
