# Supplementary material for: Placental OPRM1 DNA methylation and associations with neonatal opioid withdrawal syndrome, a pilot study
Source: Explor Med. Author manuscript; Available in PMC 2021 Mar 23. (PMC7985727; doi:10.37349/emed.2020.00009)
Supplement: Supplementary Material [file NIHMS1678955-supplement-Supplementary_Material.pdf]

Supplemental Methods

Laboratory Methods:

For DNA methylation analysis, 250 ng of extracted genomic DNA was bisulfite treated using the EZ DNA Methylation kit (Zymo Research, Inc., CA). Bisulfite treated DNA was purified according to the manufacturer's protocol and eluted to a final volume of 46 µL. PCRs were performed using 1 µL of bisulfite treated DNA and 0.2 µM of each primer. One primer was biotin-labeled and HPLC purified in order to purify the final PCR product using sepharose beads. PCR products were bound to Streptavidin Sepharose HP (GE Healthcare Life Sciences), after which the immobilized PCR products were purified, washed, denatured with a 0.2 µM NaOH solution, and rewashed using the Pyrosequencing Vacuum Prep Tool (Pyrosequencing, Qiagen), as per the manufacturer's protocol. Next, 0.5 µM of sequencing primer was annealed to the purified single-stranded PCR products. 10 µL of the PCR products were sequenced by Pyrosequencing on the PSQ96 HS System (Pyrosequencing, Qiagen) following the manufacturer's instructions.
